# Active information sampling in health and disease

**DOI:** 10.1016/j.neubiorev.2025.106197

**Published:** 2025-05-03

**Authors:** Bahaaeddin Attaallah, Pierre Petitet, Masud Husain

**Affiliations:** a Nuffield Department of Clinical Neurosciences, University of Oxford, Oxford OX3 9DU, UK; b Centre for Preventive Neurology, Queen Mary University of London, London EC1M 6BQ, UK; c Department of Experimental Psychology, University of Oxford, Oxford OX1 3PH, USA

**Keywords:** Information Gathering, Decision Making, Uncertainty, Impulsivity, Neurotransmitters, Hippocampus

## Abstract

Active information gathering is a fundamental cognitive process that enables organisms to navigate uncertainty and make adaptive decisions. Here we synthesise current knowledge on the behavioural, neural, and computational mechanisms underlying information sampling in healthy people and across several brain disorders. The role of cortical and subcortical regions spanning limbic, insular, fronto-parietal, and striatal systems is considered, along with the contributions of key neurotransmitters involving norepinephrine, dopamine, and serotonin. We also examine how various clinical conditions, including schizophrenia, obsessive-compulsive disorder, and Parkinson’s disease have an impact on information gathering behaviours. To account for the findings, we outline a neuroeconomic perspective on how the brain may evaluate the costs and benefits of acquiring information to resolve uncertainty. This work highlights how active information gathering is a crucial brain process for adaptive behaviour in healthy individuals and how its breakdown is relevant to several psychiatric and neurological conditions. The findings have important implications for developing novel computational assays as well as targeted interventions in brain disorders.

## 1 Introduction

The ability to actively gather information from the environment is crucial for adaptive decision-making and behaviour (Dey and Gottlieb, 2019; Wilson, 2000). In a world filled with uncertainty, organisms must constantly sample their surroundings to update their knowledge and guide future actions (Petitet et al., 2021; Stephens, 2008). This process of information gathering allows us to navigate complex environments, learn from experience, and make choices that maximise rewards whilst minimising risks (Gottlieb and Oudeyer, 2018; Rangel et al., 2008).

Active information sampling is distinct from passive or one-shot information processing, as it involves deliberate decisions about what information to seek, when to seek it, and how much to gather before committing to a choice (Dey and Gottlieb, 2019; Kobayashi and Kable, 2024; Petitet et al., 2021). This behaviour requires a delicate balance between exploration and exploitation – gathering enough information to make informed decisions whilst avoiding excessive sampling that may be costly in terms of time, energy, or missed opportunities. Recent years have seen growing interest in understanding the cognitive and neural mechanisms underlying active information gathering. This surge of research has been driven by several factors: 1) recognition that many psychiatric and neurological conditions involve aberrant information sampling behaviours, 2) advances in computational modelling that allow for more precise quantification of information gathering processes, and 3) new neuroimaging and electrophysiological techniques that enable investigation of the neural circuits involved.

In this review, we synthesise current knowledge on active information gathering across multiple levels of analysis from behaviour to brain systems. We begin by reviewing behavioural paradigms used to study active information gathering in humans, and then discuss its cognitive components from a neuroeconomic perspective. We then examine how various clinical conditions have an impact on information gathering, with a focus on disorders involving dopamine dysfunction (e.g., schizophrenia, Parkinson’s disease) and those characterised by compulsivity or anxiety (e.g., obsessive-compulsive disorder).

Next, we review the roles of key neurotransmitter systems, including norepinephrine, dopamine, and serotonin, in modulating information sampling behaviour. We also discuss the contributions of specific brain regions, with emphasis on the hippocampus, amygdala, insula, parietal and frontal cortices, as well as the valuation system. Throughout, we highlight how computational approaches have advanced our understanding by allowing more precise quantification of the cognitive processes involved in information gathering.

Finally, we summarise the findings outlining a neuroeconomic perspective for understanding how the brain evaluates the costs and benefits of acquiring information to resolve uncertainty. This framework synthesises findings across levels of analysis and provides insights for future research. We conclude by discussing the implications of this work for developing novel computational assays of information gathering behaviour and potential avenues for targeted interventions in clinical populations.

By examining information gathering as a transdiagnostic process relevant to multiple psychiatric and neurological conditions, this review aims to shed light on fundamental mechanisms of adaptive decision-making and behaviour. Understanding how humans and other animals navigate uncertainty through active sampling of their environment has broad implications for cognitive science, neuroscience, and clinical practice.

## 2 How to study active information gathering?

### 2.1 Behavioural paradigms

**Fig. 1:**
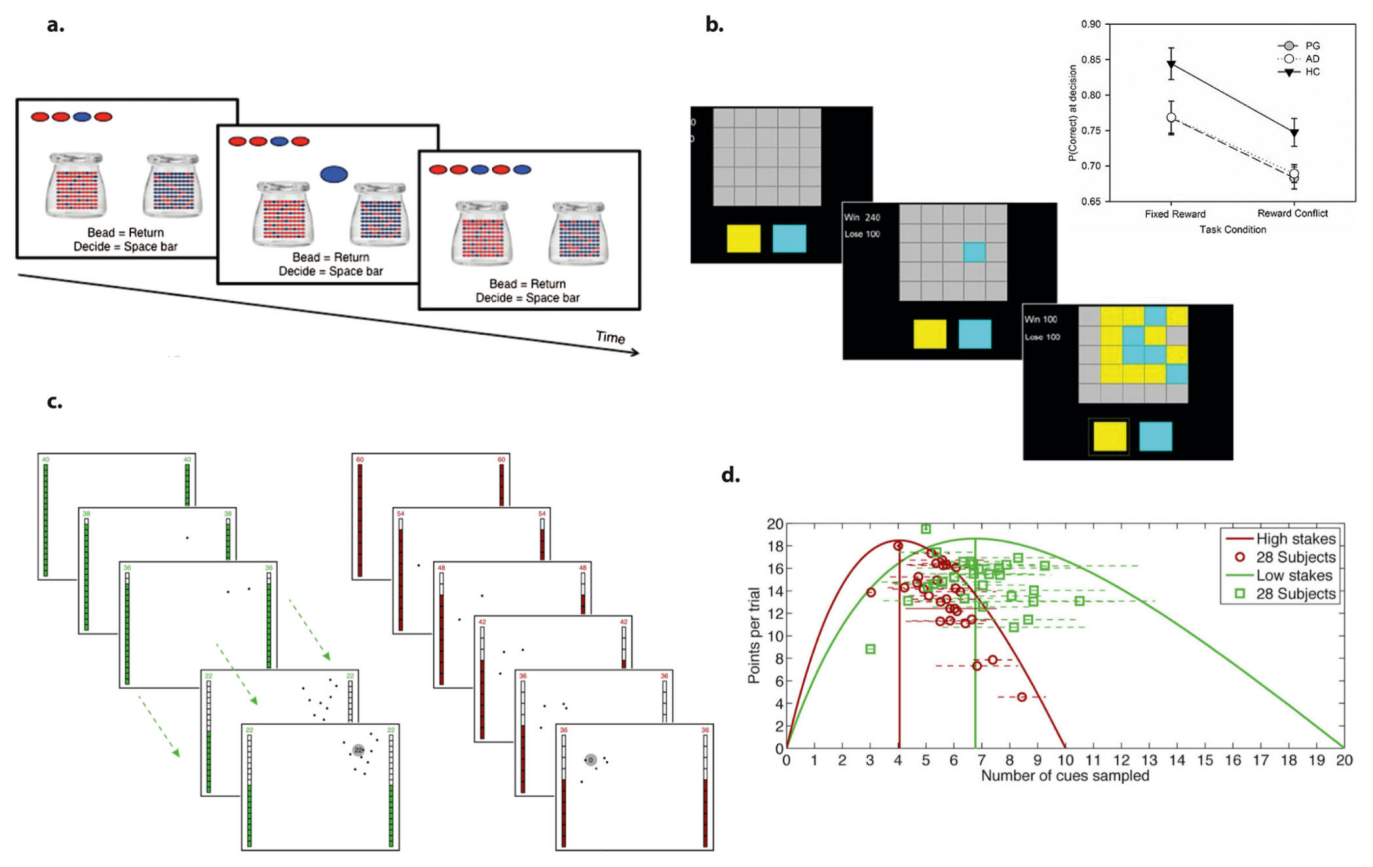
Previous behavioural paradigms investigating information gathering. a. Beads task. In this task, participants sequentially draw samples (beads) from one of two jars with different colour distributions (predominantly red or blue). Their objective is to infer from which jar the samples are being drawn (illustration from Voon et al., 2016). b. Information sampling tasks (IST) (Clark et al., 2006). This task follows a similar principle to the beads task. Participants open boxes to determine the predominant colour on the screen (blue or yellow). The task includes two conditions: one in which additional observations come at a cost (reward conflict) and another where observations are free (fixed reward). The probability of making a correct decision, P(correct), can be computed and used as a behavioural index of reflection impulsivity. For example, one study found that addiction (problem gamblers, PG) and binge drinking (AD) were associated with lower P(correct), reflecting higher impulsivity (Lawrence et al., 2009). c. Task used in (Juni et al., 2016) featuring a continuous uncertainty space and an explicit sampling cost. Participants attempt to locate a hidden target by requesting samples derived from a distribution centred around the target. A similar but more complex design was developed in the Circle Quest task (Petitet et al., 2021), where participants could freely decide when and how to sample. This allowed researchers to capture not only the total number of samples collected but also the speed and efficiency of sampling behaviour. d. Calculation of the optimal number of samples based on the information gain function, which determines the utility of samples as a function of their number and cost. Participants tended to over-sample when the cost of sampling was high (Juni et al., 2016).

Several behavioural tasks have been developed to examine active information gathering in humans. One of the earliest and most widely used is the beads (urn) task (Fig. 1a) (Phillips et al., 1966). Various versions of this task have been designed to probe different cognitive mechanisms underlying probabilistic decision-making and information sampling (Dudley et al., 2016; Garety et al., 1991; Garety et al., 2005; Moritz et al., 2007; Speechley et al., 2010; Sternheim et al., 2011; Voon et al., 2016; Westermann et al., 2012). In its standard form, participants sequentially draw beads from a jar containing two colours in predefined proportions (e.g., 40/60 or 20/80) and must infer the predominant colour. The primary behavioural measure is the number of draws before making a decision, which, when compared to an optimal solution or control performance, provides insights into information-sampling behaviour. For instance, reduced sampling—termed reflection impulsivity—has been associated with the jumping to conclusions bias in certain patient populations (Balzan et al., 2012; Colbert and Peters, 2002; Huq et al., 1988; Messer, 1976; Ross et al., 2015).

Over time, modifications have been introduced to address limitations such as memory demands and task comprehension while expanding the cognitive processes under investigation. These adaptations include changes in stimuli (e.g., the “fish task” (Speechley et al., 2010)) and manipulations of economic variables to refine the analysis of decision-making (Banca et al., 2015; Esslinger et al., 2013; Fine et al., 2007; Furl and Averbeck, 2011; Jacoby et al., 2014; Lincoln et al., 2010; Menon et al., 2008; Moritz et al., 2017; Ross et al., 2015; Westermann et al., 2012; Woodward et al., 2009). Some versions impose costs on acquiring additional samples, adjust reward contingencies for decision accuracy, or manipulate the level of uncertainty faced by participants (Banca et al., 2015; Esslinger et al., 2013; Furl and Averbeck, 2011; Ross et al., 2015; Woodward et al., 2009). The specific experimental design depends on the research question and hypothesis.

Another widely used paradigm following the same principle is the information sampling task (IST)(Fig. 1b), which has gained popularity, particularly in patient studies (Averbeck et al., 2013; Clark et al., 2006). The IST reduces visual processing and working memory demands by presenting participants with a grid of 25 boxes, each concealing one of two colours. Participants uncover boxes sequentially before deciding which colour is predominant. The accuracy probability at the point of decision quantifies the level of uncertainty at which individuals are willing to commit. The task incorporates sampling costs, creating a trade-off between the benefit of additional evidence and the cost of acquiring it.

The categorical nature of the decisions (binary or limited discrete choices) in IST and beads task constrains their ecological validity and limits their utility for economic analyses of sampling behaviour. To address this issue, Juni et al. (2016) developed a paradigm in which uncertainty is modelled as a continuous distribution, updated dynamically with additional observations (Fig. 1c). In this task, participants attempt to localise a hidden target on a screen. Clues about the target’s location—sampled at a cost—are drawn from a Gaussian distribution, allowing researchers to compute an optimal solution based on subjective utility functions.

Building on this approach, Petitet et al. (2021) introduced Circle Quest, a more sophisticated task designed to probe complex aspects of information gathering. Unlike previous paradigms, this one allows participants to control both the quantity and strategy of their sampling, enabling the measurement of sampling efficiency and speed. This refinement provides a richer framework for understanding active information gathering and decision-making under uncertainty.

These paradigms collectively provide valuable insights into the cognitive mechanisms underlying information gathering, with recent advances allowing for more nuanced investigations of uncertainty estimation, sampling strategies, and decision-making processes.

### 2.2 A neuroeconomics perspective on active sampling

Active information gathering (seeking, sampling) applies to situations where agents make deliberate decisions to sequentially obtain pieces of information (samples) to resolve uncertainty (Dey and Gottlieb, 2019; Gantz et al., 2016; Gottlieb and Oudeyer, 2018; Nwone and Mutula, 2020; Petitet et al., 2021; Wilson, 2000). This is crucially distinct from passive information gathering, where agents passively process stimuli without explicitly deciding to acquire information (Nwone and Mutula, 2020). In passive gathering, agents’ decisions are often limited to whether they continue accepting the information flow from a certain source, rather than actively interacting with the environment to choose which sources to consult and how to obtain information. This highlights the importance of agency – the ability to control and direct one’s information intake – in ecological settings.

**Fig. 2:**
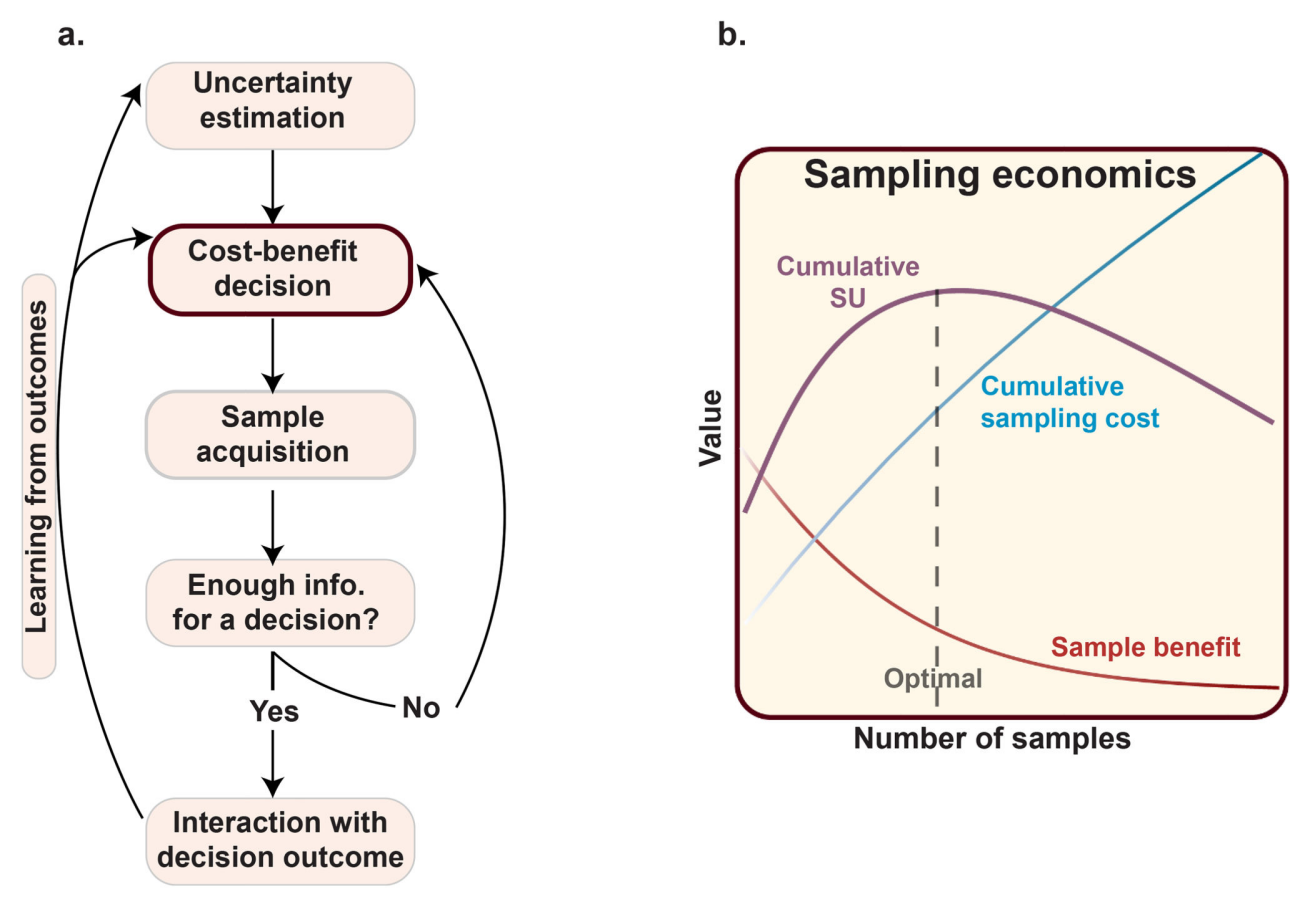
Active information gathering framework. a Behavioural components of active information gathering. At each step, as agents acquire information, they estimate the uncertainty within their environment while making a goal-directed decision. To determine whether obtaining an additional sample is worthwhile, agents conduct a cost-benefit analysis, weighing the cost of acquiring new information against its expected benefit—specifically, how much the new information is likely to improve their decision and lead to a better outcome. If the benefit of sampling outweighs its cost, the agent proceeds to acquire the sample. This process continues iteratively until the cost of obtaining further samples exceeds their expected benefit. At this point, the agent stops sampling and makes a final decision, leading to an outcome. The agent then interacts with this outcome, consuming the expected reward, comparing it with prior expectations, and using this feedback to refine future decision-making. b This trade-off between information gain and sampling cost dictates an optimal stopping point, where the subjective utility (SU) of the decision is maximised. The inset illustrates the evolution of SU across successive samples, highlighting the diminishing benefit of additional information alongside increasing cumulative costs. The optimal number of samples is marked at the peak of cumulative SU, where uncertainty reduction is maximally balanced against the cost of further sampling.

Active sampling can be conceptualised as a dynamic, multi-stage process by which agents reduce uncertainty and optimise decision-making. These steps are schematically depicted in Fig. 2. The process commences with the assessment of environmental uncertainty, wherein agents form subjective estimates by mapping the available information onto their internal representations (Daunizeau et al., 2010; Grupe and Nitschke, 2013; Pulcu and Browning, 2019). For example, when a buyer encounters an online marketplace with no prior knowledge of the options available, uncertainty is maximal, and all alternatives are initially assigned equal value. In contrast, prior experiences can lower subjective uncertainty, thereby differentiating the perceived value of each option.

Next, agents assign a subjective value to information, based on its instrumental value – its capacity to reduce uncertainty. This value reflects the impact of new data on refining internal models and diminishing uncertainty to inform subsequent actions and decisions. However, information may also possess inherent hedonic or cognitive values, influencing agents’ affect and cognition, respectively (Gottlieb and Oudeyer, 2018; Sharot and Sunstein, 2020). The total subjective value of information is ultimately weighed against the cost of acquisition (e.g,. time, effort, cognitive resources, or financial expenses) (Clark et al., 2006; Jones et al., 2019; Juni et al., 2016; Petitet et al., 2021)

In such a conceptualisation, the decision to acquire information is governed by a cost–benefit analysis, where agents compare the expected utility of information against the cost of sampling. This evaluation is dynamic, with each new piece of evidence informing updated utility estimates and further decisions about whether additional sampling is warranted (Fig. 2b.).

In goal-directed instrumental sampling, an optimal stopping rule determines when sampling should cease. This occurs when the marginal benefit of acquiring more information no longer justifies its cost, leading the agent to commit to a decision (Clark et al., 2006; Jones et al., 2019; Juni et al., 2016; Phillips et al., 1966). The nature of these costs and their accumulation can vary across contexts, shaping different strategies for information gathering. Fig. 2b. depicts one example of how such computations evolve dynamically over time with successive sampling.

Finally, once a decision is made, agents transition to goal interaction and outcome evaluation. This phase includes comparing expected and actual outcomes, generating prediction errors that refine future decision-making and learning (Den Ouden et al., 2012; Glimcher, 2011; Schultz, 2016, 2017). Notably, while this outcome-based learning occurs post-decision, the earlier information sampling phase is better characterised by inferential processes to guide active sampling decisions and draw conclusions to reduce uncertainty.

In conclusion, active information gathering is a complex behavioural process involving several cognitive components that drive sampling decisions. These components involve estimating uncertainty, performing cost-benefit valuations, interacting with goals and learning from outcomes.

## 3 Information gathering across different conditions

### 3.1 Healthy humans

Over the past decades, numerous studies have examined how healthy individuals process and gather information (Battaglia and Schrater, 2007; Busemeyer and Rapoport, 1988; Faisal and Wolpert, 2009; Ferguson, 1989; Hau et al., 2008; Hertwig et al., 2004; Jones et al., 2019; Juni et al., 2016; Tversky and Edwards, 1966). A key question in this research is whether humans tend to over- or under-sample information, with a focus on characterising rational behaviour through cost-benefit analyses. Over-sampling occurs when an individual gathers more information than would be expected from an economically optimal perspective—continuing to sample even when the cost of acquiring additional evidence outweighs its expected benefit.

Empirical findings suggest that human sampling behaviour varies depending on the task environment. Early work by Tversky and Edwards (1966) demonstrated that participants often acquire more information than necessary compared to an optimal observer. In their study, participants observed sequences of binary cues (light on/off) to infer un-derlying probabilities, choosing at each stage whether to continue sampling or make a prediction. This over-sampling behaviour was interpreted as a form of ‘conservatism effect’—where new evidence had a weaker-than-expected influence on prior beliefs, deviating from Bayesian updating (Phillips et al., 1966).

Conversely, other studies have reported under-sampling behaviour in different experimental paradigms (Hau et al., 2008; Hertwig et al., 2004). For instance, in a card-sampling task where participants drew from different decks to determine which was most profitable, participants significantly under-sampled before making a choice (Hertwig et al., 2004). One explanation for these contrasting findings lies in the specific task conditions under which information gathering occurs. When explicit costs are associated with sampling—such as in the beads, information sampling task (IST), and dart paradigms—participants often exhibit over-sampling, particularly when costs are high (Jones et al., 2019; Juni et al., 2016). In contrast, when sampling costs are minimal or absent, under-sampling behaviour is more common (Attaallah et al., 2022; Attaallah et al., 2024; Juni et al., 2016).

Importantly, such behaviours may not necessarily reflect sub-optimality, but instead may be understood through alternative normative frameworks. A prominent account suggests that individuals seek to maximise reward rate—the amount of reward gained per unit time-—rather than optimising accuracy or expected utility on each trial (Bogacz et al., 2006; Bogacz et al., 2010). From this perspective, sampling incurs not only explicit costs but also opportunity costs, whereby time spent gathering information could reduce the overall reward rate. Thus, early termination of sampling may reflect a rational trade-off between decision accuracy and time efficiency, particularly under conditions where rewards are time-sensitive.

In addition, decision-making during information gathering is subject to several latent or subjective costs that shape behaviour beyond task-defined incentives. These include affective and cognitive costs, such as boredom, fatigue, or attentional demands, which may accumulate over time and promote early commitment (Lorist et al., 2000; Mullette-Gillman et al., 2015; Petitet et al., 2021). Even when sampling is nominally cost-free, participants may limit evidence acquisition to avoid such internal burdens. Likewise, non-instrumental benefits, such as the hedonic value of knowing or curiosity-driven exploration, may bias behaviour toward over-sampling, particularly in contexts where information itself is rewarding (Kelly and Sharot, 2021; Sharot and Sunstein, 2020).

As the cognitive demands of a task increase—such as when individuals must integrate complex probabilistic information or adapt to dynamic environments—the computational cost of maintaining and updating internal models can itself become a significant deterrent to extended sampling (Petitet et al., 2021). This internal burden does not act in isolation; it often coincides with rising urgency signals, which are thought to increase over time and reduce the threshold for commitment (Cisek et al., 2009; Thura et al., 2012). Together, these factors exert converging pressure toward early decision-making: the growing effort required for continued inference combines with a temporal drive to act, resulting in premature choices even when uncertainty remains. In this way, computational load and urgency interact to shape the dynamics of sampling, particularly under time constraints or when opportunity costs are perceived to be high.

Overall, information gathering is governed by a complex integration of multiple cost domains—including temporal, cognitive, affective, and computational—intertwined with evolving estimates of uncertainty, subjective confidence and sampling benefits. These costs are not necessarily static or accurately perceived, and may give rise to systematic biases in sampling, such as premature stopping or compulsive over-sampling. As such, human information gathering reflects not only normative optimisation but also bounded rationality, shaped by latent internal constraints and contextual demands.

### 3.2 Delusional spectrum

The earliest exploration of the clinical implications of deficits in information gathering centred on delusional disorders, particularly schizophrenia. In a seminal study, Huq et al. (1988) employed the Beads task to compare the information-gathering behaviour of individuals with schizophrenic delusions to that of healthy controls. Their findings revealed that delusional patients sampled significantly less information than both healthy individuals and non-delusional patients before making decisions. This phenomenon, widely known as the “jumping to conclusions” (JTC) bias, reflects a tendency to reach decisions prematurely, without sufficient evidence.

Interestingly, the JTC bias is not exclusive to schizophrenia. It has also been observed in non-schizophrenic individuals with delusions (McLean et al., 2016) and in healthy individuals prone to delusional thinking (Balzan et al., 2012; Colbert and Peters, 2002; Rausch et al., 2016; Ross et al., 2015). These findings support a dimensional perspective on JTC bias, suggesting that it represents a trait-like vulnerability rather than a state-dependent deficit. Nevertheless, lon-gitudinal research is still required to establish whether the presence of JTC bias in otherwise healthy individuals confers an increased risk for the later development of delusions and psychosis. Moreover, emerging evidence indicates that the intensity of the JTC bias may be modulated by emotional salience: reasoning biases tend to be more pronounced in emotionally charged or self-relevant contexts (Romero-Ferreiro et al., 2022; Warman and Martin, 2006). This highlights the potential role of affective processes in lowering the threshold for evidence accumulation and suggests possible interactions with affective dysfunction, such as that observed in mood or anxiety disorders.

Several explanations have been proposed for the association between delusional disorders and the JTC bias. One account focuses on cognitive rigidity in belief formation. Research suggests that limited belief flexibility may underlie the link between reduced sampling and delusions (Garety et al., 2005). Patients with delusions often exhibit a bias against disconfirmatory evidence (BADE), whereby they discount or ignore evidence that contradicts their pre-existing beliefs (Moritz and Woodward, 2006). This rigidity in belief updating could explain why delusional patients gather less information before forming conclusions. However, more recent findings indicate that belief flexibility, under-sampling, and delusions are independent constructs, challenging the notion of a direct causal relationship between them (So et al., 2012). A second account emphasises aberrant cost-benefit evaluations in psychosis. Here, JTC is viewed as a consequence of distorted internal representations of the subjective costs and benefits of sampling (Ermakova et al., 2019). Individuals with delusions may undervalue the potential utility of additional evidence, or conversely, overestimate the effort or time cost of continued sampling. Supporting this interpretation, computational modelling studies have demonstrated that patients with early psychosis exhibit lower decision thresholds, committing to choices with less certainty than controls, even when there are no explicit penalties for prolonged sampling (Bentall and Swarbrick, 2003; Broome et al., 2007; Moritz et al., 2007). These findings suggest that patients may encode intrinsic costs to information gathering. Recent evidence further links such cost over-estimation to negative symptoms, particularly apathy, implicating motivational

dysfunction as a contributing factor (Moran et al., 2023).

Related to this account is the hypersalience of evidence hypothesis, which proposes that delusion-prone individuals attribute excessive weight to early pieces of information, resulting in rapid confidence accumulation and belief fixation (Speechley et al., 2010). In this case, additional sampling is perceived as redundant because the individual subjectively feels they already possess sufficient evidence.

Notably, the degree of decision noise—random variability in how evidence is integrated—appears to differ across stages of illness. While noise plays a significant role in chronic schizophrenia (Moutoussis et al., 2011), patients in the early stages of psychosis are more sensitive to cost manipulations, pointing toward aberrant valuation processes rather than global cognitive impairment as the key driver of JTC bias (Ermakova et al., 2019; Moutoussis et al., 2011). This distinction aligns with broader models of schizophrenia that propose intact basic inference mechanisms, but maladaptive assignment of salience or cost.

A third explanatory account focuses on metacognitive dysfunction, defined as impairment in the monitoring and regulation of one’s own cognitive processes. While earlier theories suggested that JTC might arise from executive deficits or task miscomprehension (Lunt et al., 2012), these have largely been ruled out by studies showing that patients with schizophrenia understand task instructions and can reason about probabilities comparably to controls (Ermakova et al., 2019; Fine et al., 2007). Nonetheless, metacognition encompasses multiple dimensions, including self-monitoring (awareness of cognitive performance), self-reflection (evaluating and updating beliefs), and insight (recognition of cognitive biases or distortions) (Fleming, 2024). Deficits in each of these domains have been documented in schizophrenia (Lysaker et al., 2015). Several studies have found that metacognitive performance correlates with information sampling behaviour in healthy populations and delusional spectrum (Buck et al., 2012; Desender et al., 2018; Guigon et al., 2024; Schulz et al., 2023). Interventions designed to reduce the JTC bias have been associated with improvements in meta-cognitive functioning and decision-making capacity (Naughton et al., 2012; Turner et al., 2018). These findings suggest that individuals may not only misrepresent the cost of continued sampling but also lack the metacognitive capacity to appraise the quality of their decisions or recognise bias. In such cases, JTC may reflect a breakdown in self-regulation, whereby the agent lacks access to internal signals that would otherwise guide continued deliberation. Further studies are needed to clarify the relationship between specific metacognitive dimensions and active information sampling components in delusional reasoning, and to determine whether deficits in insight, uncertainty monitoring, or self-evaluation make distinct contributions to the JTC phenotype.

Overall, while multiple cognitive and decision-making mechanisms have been implicated in the JTC bias, including belief inflexibility, cost misestimation, intolerance of uncertainty, and metacognitive dysfunction, further research is required to disentangle their relative contributions and clarify their role in the emergence and persistence of delusions.

### 3.3 Impulsivity

Impulsivity is clinically defined as a predisposition to act rapidly and without sufficient forethought or consideration of the potential adverse consequences (Moeller et al., 2001). One particular manifestation, known as reflection impulsivity, refers to hasty decision-making in the context of information gathering, whereby decisions are reached with minimal supportive evidence—an effect that is equivalent to the “jumping to conclusions” (JTC) bias described in schizophrenia.

A number of studies have investigated information gathering and decision-making under uncertainty in the context of impulsivity (Averbeck et al., 2013; Delazer et al., 2012; de Rezende Costa et al., 2016; Djamshidian et al., 2012; Kagan et al., 1964; Lees et al., 2013; Lunt et al., 2012; Snorrason et al., 2011; Voon et al., 2017). In these reports, reflection impulsivity is typically operationalised as under-sampling of evidence prior to making a decision (Messer, 1976). Early investigations often utilised the Matching Familiar Figures Test (MFFT) (Kagan, 1966; Kagan et al., 1964), where participants (typically children) are presented with a target figure and then required to select the matching figure from an array of similar figures differing in one or several attributes. Although the MFFT is not a dedicated information gathering task per se, it has been widely used as an indirect measure of reflection impulsivity. This is because performance on the MFFT is assessed based on response time and accuracy, with faster decisions and higher error rates serving as indicators of reflection impulsivity, reflecting a tendency to make decisions with insufficient evidence. This approach is distinct from the assessment of motor impulsivity, which pertains to the inability to stop an ongoing process (e.g., as measured by stop-signal or Go/No-Go paradigms) (Logan et al., 2016), and from waiting impulsivity, which is the inability to delay an action, typically assessed through delay discounting tasks (Dalley et al., 2011; Voon et al., 2014).

While not a separate disorder, reflection impulsivity is a prominent transdiagnostic cognitive feature observed in a range of neurological and psychiatric disorders, including Parkinson’s disease (PD) (Averbeck et al., 2013; Delazer et al., 2012; de Rezende Costa et al., 2016; Djamshidian et al., 2012; Kehagia et al., 2014; Lees et al., 2013), frontal lobe lesions (Lunt et al., 2012), addiction (Banca et al., 2015; Clark et al., 2006; Irvine et al., 2013; Joos et al., 2013; Mishra et al., 2010; Stevens et al., 2015) and attention deficit hyperactivity disorder (ADHD) (Stevens et al., 2015). In PD, reflection impulsivity is particularly evident in patients with impulse control disorders (ICD), suggesting that excess dopamine—often introduced through dopamine agonists or replacement therapy—may promote under-sampling (Averbeck et al., 2013; Djamshidian et al., 2012). Yet, the observation of reflection impulsivity in drug-naïve PD patients (de Rezende Costa et al., 2016), alongside reports of PD patients on L-dopa performing comparably to healthy controls (Djamshidian et al., 2012), challenges the notion that dopamine is the sole contributor. These conflicting findings underscore the complexity of impulsivity in PD, implicating additional factors such as a bias towards novel stimuli (Averbeck et al., 2013; Djamshidian et al., 2011), altered temporal discounting (Averbeck et al., 2013; Joutsa et al., 2015), potential associations with apathy (Sinha et al., 2013), and disruptions within cortico-striatal networks (Cilia and Van Eimeren, 2011; van Eimeren et al., 2010).

Transdiagnostic analyses have further delineated the features of reflection impulsivity across disorders (Averbeck et al., 2013; Djamshidian et al., 2012). For example, one study compared PD patients with and without ICD to illicit drug users and pathological gamblers (Djamshidian et al., 2012). The findings revealed that PD patients with ICD exhibited sampling behaviour similar to that of illicit drug users—sampling significantly less evidence than controls under all conditions—whereas PD patients without ICD displayed a pattern more akin to pathological gamblers, sampling less than controls only under conditions of high uncertainty. These distinctive patterns may reflect divergent underlying mechanisms: a generalised reduced sensitivity to uncertainty in PD with ICD versus diminished flexibility in response to fluctuating uncertainty in PD without ICD. Computational modelling of behavioural data further suggests that reflection impulsivity in PD with ICD may be linked to uncertainty regarding the mapping of future actions onto rewards (Averbeck et al., 2013). This parameter appears to be shared with other behaviours in PD with ICD, such as a bias towards novelty and altered temporal discounting, implying that various forms of impulsivity might share a common cognitive mechanism.

Whether the features or mechanisms underlying reflection impulsivity (i.e., under-sampling) in Parkinson’s disease (PD) are shared across different conditions remains under investigation. For example, a wealth of evidence has highlighted the relationship between impulsivity and addiction using both self-reported measures (Frydman et al., 2020) and objective task parameters– specifically, under-sampling on information gathering tasks (Banca et al., 2015; Clark et al., 2006; Irvine et al., 2013; Joos et al., 2013; Mishra et al., 2010; Stevens et al., 2015).

Crucially, reflection impulsivity appears to be a prevalent feature across various types of addictive behaviour, rather than being specific to the substance or nature of the addiction. It has been reported in alcohol dependence (Joos et al., 2013), binge drinking (Banca et al., 2016), pathological gambling (Mishra et al., 2010) and gaming (Irvine et al., 2013), as well as in substance abuse involving cocaine, opioids and amphetamines (Clark et al., 2006; Stevens et al., 2015). Notably, under-sampling seems to be a stable feature in these conditions, since individuals who recover from addiction continue to exhibit impulsivity (Clark et al., 2006), suggesting that it may serve as a vulnerability marker in otherwise healthy people (Verdejo-García et al., 2008). Mechanistically, under-sampling in addiction may be linked to a hypersensitivity to reward, which enhances the valuation of uncertain options that offer higher rewards. Several studies have demonstrated that drug users exhibit an increased sensitivity to reward when shaping preferences and subjective valuation (Verdejo-Garcia et al., 2018).

Overall, a single unifying mechanism for under-sampling in reflection impulsivity has yet to be established. In some conditions, such as PD, it may be associated with a lower sensitivity to uncertainty relative to reward, whereas in addiction heightened reward sensitivity may play a more central role. A computational account that balances the competing processes of reward maximisation and uncertainty minimisation may ultimately provide crucial insights into the mechanisms underlying reflection impulsivity and its associated clinical conditions.

### 3.4 Obsessive compulsive disorder (OCD)

OCD is characterised by intrusive thoughts or urges (obsessions) that often lead to repetitive behaviours (compulsions) aimed at reducing the distress associated with these obsessions (Stein et al., 2019). In contrast to delusional disorders or impulsivity –conditions typically associated with under-sampling– OCD is frequently linked to over-sampling (R. Dar, 2004; Fear and Healy, 1997; Foa et al., 2003; Hauser et al., 2017; Pelissier and O’connor, 2002; Stern et al., 2013; Toffolo et al., 2013) (although see Frydman et al. 2020, Grassi et al. 2015, Jacobsen et al. 2012, Morein-Zamir et al. 2020, and Voon et al. 2017). Patients with higher compulsivity scores tend to seek more information, particularly when the cost is low (Banca et al., 2015; Hauser et al., 2017). While some studies have shown that reward incentives can modulate this behaviour to improve outcomes, other reports suggest that over-sampling in OCD may reflect a form of *cognitive rigidity*, rendering it resistant to reward manipulations (Chamberlain et al., 2007).

Several mechanisms have been proposed to explain over-sampling in OCD. For instance, some studies suggest that OCD patients may exhibit a metacognitive deficit in subjective uncertainty estimation, rating themselves as more uncertain than controls for equivalent levels of objective uncertainty (R. Dar, 2004; Stern et al., 2013).More recently, research has indicated a dissociation between confidence and action in OCD, whereby patients update their confidence in response to new evidence similarly to controls but fail to utilise this updated information to optimise their actions (Vaghi et al., 2017). These findings have been replicated using a transdiagnostic approach that extracted a compulsivity dimension from a large sample of healthy participants performing the task online (Seow and Gillan, 2020). Such observations suggest a ‘stuck in habit’ phenomenon in OCD, leading to purposeless sampling and a disregard for both the acquired knowledge and its associated costs (Gillan et al., 2014). An alternative account posits that over-sampling in OCD may result from a delayed emergence of cost signals and associated urgency, manifesting as a higher decision threshold to terminate information gathering (Hauser et al., 2017; Hauser et al., 2018) –a pattern opposite to that observed in delusional patients, who tend to assign higher costs to sampling.

Conversely, some investigations have reported that OCD patients under-sample and make hasty decisions, thereby implicating an element of impulsivity (e.g., impulsive compulsions) in the condition (Grassi et al., 2015; Jacobsen et al., 2012; Voon et al., 2017). This inconsistency regarding whether OCD patients over- or under-sample highlights two important considerations when examining information gathering and goal-directed behaviour in OCD. First, as noted by previous reports (Morein-Zamir et al., 2020), the methods commonly used (predominantly the beads task) may lack the specificity required to capture the underlying mechanistic components accurately. Second, given the significant overlap between OCD and other neuropsychiatric disorders (e. g., impulsivity and anxiety), careful phenotyping of the condition or adopting a transdiagnostic approach is essential for making reliable advances.

### 3.5 Affective disorders: anxiety and depression

Deficits in processing uncertainty have been consistently regarded as a key mechanism in anxiety spectrum disorders (Bishop and Gagne, 2018; Boswell et al., 2013; Carleton et al., 2012; Grupe and Nitschke, 2013; Gu et al., 2020; C. A. Hartley and Phelps, 2012; Hildebrand-Saints and Weary, 1989; Pulcu and Browning, 2019; Saulnier et al., 2019). These deficits may manifest at various stages of goal-directed behaviour, influencing both value-based decision-making and information gathering. One prominent framework conceptualises anxiety as an anticipatory reaction to uncertainty regarding future aversive or threatening events (Grupe and Nitschke, 2013). According to this model, anxiety arises from an inflated estimation of uncertainty and its associated costs, leading to an overestimation of the likelihood of negative events and to exaggerated anticipatory responses and attention. This overestimation, combined with hyper-vigilant responses, can set in motion a vicious cycle that impairs safety learning and fosters dysfunctional associations between environmental cues and negative outcomes. Consequently, individuals may find it difficult to form an accurate representation of their environment (Grupe and Nitschke, 2013).

In line with this anticipatory model of anxiety, which emphasises future-oriented emotional states, the behavioural inhibition system (BIS) similarly posits that the perception or expectation of uncertainty drives both behavioural and physiological responses to perceived threats (McNaughton, 1982). According to this model, anxiety is considered a function of a septo-hippocampal system that acts as a comparator between expectations and observations, thereby triggering behavioural inhibition that leads to the avoidance of aversive cues, such as uncertainty.

Based on these models, one would expect anxious individuals to exhibit two seemingly contrasting information sampling behaviours in response to their hypersensitivity to uncertainty: (i) they might collect as much information as possible (i.e., over-sample) to reduce uncertainty before making decisions, or (ii) they may display pronounced behavioural inhibition, resulting in the total avoidance of uncertainty. The drivers of these behavioural responses could include an exaggerated estimation of uncertainty, the assignment of greater weight to uncertain outcomes, or a perceived lower cost associated with acquiring additional samples when gathering information (Grupe and Nitschke, 2013).

Only a few studies have investigated information gathering in anxiety and related disorders (Jacoby et al., 2014; Sternheim et al., 2011; Volans, 1976). The findings from these reports are inconsistent. For example, one study found that participants with anxiety did not differ from healthy controls in their information gathering, even though the anxious individuals reported greater distress during task performance and a higher intolerance to uncertainty on self-report measures (Jacoby et al., 2014). Although this might suggest that information gathering remains intact in anxiety—and potentially support the delineation of anxiety from disorders once grouped within its spectrum, such as OCD (Thomas Widiger et al., 1994)—a more fine-grained investigation is required to substantiate this claim and to explore how intolerance of uncertainty manifests during information gathering.

Depression shares several characteristics with anxiety regarding their influence on motivated decision-making and the processing of uncertainty (Bishop and Gagne, 2018). Both conditions have been associated with an increased intolerance to uncertainty (K. A. Dar et al., 2017; Dugas, 1997; Freeston et al., 1994), impaired learning in probabilistic environments (Gagne et al., 2020), and reduced reward valuation (Ng et al., 2019; Winer and Salem, 2016).

Research into the influence of depression on active information gathering is limited. Studies in social psychology have shown that depression is linked to enhanced information-seeking behaviour in social contexts, such as when individuals are required to ask questions in an interview setting (Hildebrand-Saints and Weary, 1989). Similarly, the severity of depression—as indexed by Beck’s Depression Inventory (BDI)—has been associated with extensive sampling in the Information Sampling Task (IST) (Crockett et al., 2012). It has been suggested that this over-preparation behaviour may stem from worry arising from a perceived lack of control over future events (Abramson et al., 1978; Barlow, 1991). A substantial body of literature emphasises that perceptions of and reactions to controllability over possible outcomes constitute a crucial mechanism in affective disorders, as illustrated by the learned helplessness model of depression (Liu et al., 2015). Furthermore, decreased reward valuation observed in depression may affect the cost-benefit computations underlying information sampling—depressed individuals might over-sample because of reduced sensitivity to the costs of sampling and its impact on potential rewards (Ng et al., 2019; Winer and Salem, 2016).

Affective disorders often co-occur with other clinical conditions, including psychiatric illnesses and degenerative diseases, highlighting their complex interplay with various health challenges (Bandelow and Michaelis, 2015; Malhi and Mann, 2018; Penninx et al., 2021). One notable example is subjective cognitive impairment (SCI), a condition increasingly recognized in ageing populations (Jessen et al., 2020; Jessen et al., 2014; Reid and MacLullich, 2006). SCI is defined by subjective cognitive complaints without objective clinical evidence and holds significant importance in dementia research as it is considered a preclinical stage of Alzheimer’s disease (Jessen et al., 2020; Jessen et al., 2014; Reid and MacLullich, 2006). Emerging evidence suggests that affective dysfunctions, such as depression and anxiety, are central features of SCI and may underlie many of its clinical manifestations (Hill et al., 2016; Hohman et al., 2011; Pavisic et al., 2021; Reid and MacLullich, 2006). Recently, we identified deficits in information gathering as a potential mechanism linked to the affective dysfunction observed in SCI. Specifically, SCI participants demonstrated a tendency to over-sample information compared to controls and exhibited faster sampling rates. This behaviour suggests an urgency to resolve uncertainty regardless of the cost of sampling (Attaallah et al., 2022).

Overall, it seems that anxiety and depression might be both associated with over-sampling due to uncertainty intolerance.

### 3.6 Neurodevelopment and neurodegeneration

Previous research suggests that age plays a significant role in shaping information-gathering behaviour in humans. For instance, Jones et al. (2019) demonstrated that the ability to sample information efficiently approaches adult-like levels in children between the ages of six and 11. A more recent study found that children, compared to adolescents, assign lower subjective costs to information and therefore engage in more extensive sampling when information is freely available (Bowler et al., 2021). This behaviour may have an evolutionary advantage, as it fosters curiosity and learning during early development. However, other studies suggest that adolescents may tolerate uncertainty more than both younger children and adults, leading to less sampling before making decisions and following a U-shaped developmental trajectory (Van Den Bos and Hertwig, 2017). These findings imply that the ability to balance the costs and benefits of acquiring information to resolve uncertainty and make decisions begins developing early in life and stabilizes during adulthood. Despite these insights, how information-gathering behaviour evolves across the lifespan—from early childhood to later stages of life—remains poorly understood. To date, no studies have comprehensively tracked this trajectory or conducted a comparative analysis across all age groups.

Studies in older adults have shown that ageing is associated with impaired uncertainty processing and estimation, which adversely affects decision-making and learning under uncertain conditions (Nassar et al., 2016; Pietschmann et al., 2011). Similarly, research indicates that older adults are less averse to uncertainty and ambiguity, often committing to decisions with insufficient information (A. Sproten et al., 2010; A. N. Sproten et al., 2018), potentially reflecting a tendency towards under-sampling. In the context of Alzheimer’s disease (AD) – the incidence of which increases with age – studies employing information sampling tasks reveal that AD patients gather significantly less information before making decisions and tend to take riskier choices compared to controls (Sinz et al., 2008; Zamarian et al., 2015). However, the relationship between these findings and memory complaints in both healthy ageing and disease remains unclear. Further investigation is required to explore information-gathering behaviour across the AD spectrum and its connection to memory complaints in ageing, including conditions such as mild cognitive impairment (MCI) and SCI.

These findings collectively highlight how information-gathering behaviour evolves and shifts across the lifespan, shifting from extensive sampling in childhood to under-sampling in older age, with important implications for decision-making and memory complaints in health and disease.

## 4 Neuromodulation of information gathering

### 4.1 Norepinephrine

Norepinephrine (NE), a monoamine neurotransmitter, plays a significant role in modulating information gathering. Evidence for this role emerges from both clinical and experimental studies, highlighting its influence on decision-making processes and behavioural responses to uncertainty.

Clinical observations provide indirect evidence of NE’s involvement in information gathering. For instance, NE-modulating agents have occasionally been explored as adjuncts in manageing disorders characterised by excessive sampling behaviours, such as obsessive-compulsive disorder (OCD) and anxiety (Brudkowska et al., 2018). This suggests a potential role for NE in regulating the balance between over- and under-sampling of information in certain psychiatric conditions. Additionally, NE is thought to contribute to the hippocampus-centred behavioural inhibition system (BIS), which underlies anxiety and sensitivity to aversive stimuli. Within this framework, NE is proposed to modulate a neurophysiological gate that relays signals about uncertainty and threat between the hippocampus and broader limbic circuits (McNaughton, 1982). Experimental studies further support NE’s role in information gathering. One report demonstrated that blocking NE reduces the amount of information gathered before making decisions. This effect is likely due to an increase in urgency signals, which reflect heightened costs associated with prolonged sampling (Hauser et al., 2018). Conversely, atomoxetine, a drug that inhibits NE reuptake in the prefrontal cortex, has been shown to improve reflection impulsivity by reducing under-sampling in patients with Parkinson’s disease (Kehagia et al., 2014).

The mechanisms underlying NE’s effects on information gathering may relate to its established roles in arousal and attention. By promoting task engagement and careful strategies, NE facilitates more deliberate decision-making (Sara and Bouret, 2012). Phasic activity of central NE systems originating from the locus coeruleus may play a key role here, linking to brain regions such as the orbitofrontal cortex (OFC) and anterior cingulate cortex (ACC), which are involved in decision-making and task optimization (Aston-Jones and Cohen, 2005; Yu and Dayan, 2005).

Alternatively, NE might contribute to representing uncertainty and modulating physiological responses to it. This role has been demonstrated across various studies using neuroimaging (Payzan-LeNestour et al., 2013), pharmacological manipulations (Lawson et al., 2021; Marshall et al., 2016), and physiological recordings of neural activity and pupillary dynamics in response to uncertain stimuli (Dayan and Yu, 2006).

In summary, norepinephrine appears to be a critical promoter of information-gathering behaviour. Its influence likely stems from its dual contributions: representing uncertainty and regulating the costs associated with overcoming it. These insights underscore NE’s pivotal role in adaptive decision-making processes.

### 4.2 Dopamine

The observation that information gathering is affected in several clinical conditions involving dopamine dysregulation (e.g., schizo-phrenia, Parkinson’s disease PD with or without impulse control disorders ICD) suggests that dopamine may play a significant regulatory role in this behaviour. However, direct investigations of this hypothesis have yielded conflicting findings. For instance, pharmacological modulation of dopamine levels using agonists and antagonists has generally shown no significant effect on sampling behaviour in both healthy individuals and those with clinical conditions (Andreou et al., 2015; Hauser et al., 2018; Menon et al., 2008; Voon et al., 2016) (but see Lees et al. 2013). These discrepancies may be explained by the nature of the information people are motivated to gather.

Recent research provides a more nuanced understanding by highlighting dopamine’s specific role in non-instrumental information gathering—particularly when the information possesses hedonic value. For example, L-DOPA, a dopamine precursor, was found to blunt the preference for seeking additional information about potential rewards compared to losses, a preference otherwise observed in placebo groups (Vellani et al., 2020). This finding aligns with extensive literature implicating dopamine in reward processing and valuation. Dopamine has been shown to modulate the motivational salience of rewards and guide behaviour toward reward-related stimuli (Bromberg-Martin et al., 2010; Kurniawan et al., 2011; Rogers, 2011). In this context, non-instrumental information gathering—seeking information not directly tied to immediate decision-making but driven by curiosity or the hedonic value of learning may be particularly sensitive to dopaminergic signaling.

It it likely that instrumental information sampling is affected by these hedonic features and dopamine modulated aspects of information. Further research into the cost-benefit evaluation underlying information gathering could help clarify dopamine’s role in this behaviour. Specifically, examining how dopamine modulates the trade-off between the effort or cost required to obtain information and its perceived value whether instrumental or hedonic might provide deeper insights into its regulatory function. Such investigations could also help reconcile conflicting findings by accounting for task-specific factors and individual differences in dopaminergic functioning.

### 4.3 Serotonin

Over the past decade, serotonin has been increasingly implicated in information gathering. For instance, acute depletion of tryptophan, a precursor of serotonin, has been shown to promote extended sampling by diminishing the aversive impact of sampling costs (Crockett et al., 2012). Consistent with this, citalopram—an agent that increases brain serotonin by selectively inhibiting its reuptake—has been found to decrease the expected utility of choices after sampling, potentially due to a heightened perception of information cost (Livermore et al., 2021). Recent evidence also suggests that serotonin may shape how individuals learn from positive and negative outcomes. Michely et al. (2022) demonstrated that prolonged SSRI administration in healthy individuals enhances learning from punishment and reduces learning from reward, thereby biasing behaviour toward avoidance based on cumulative negative feedback. Rather than altering sampling costs directly, these findings imply that serotonin modulates the valuation of outcomes during learning and may discourage information-seeking when potential losses are salient. Collectively, this aligns with serotonin’s broader role in aversive prediction, behavioural inhibition, and adaptive responses to environmental threat (Dayan and Huys, 2009). If serotonin amplifies the perceived aversiveness of sampling costs, it follows that increased serotonin levels would promote under-sampling.

In summary, multiple neurotransmitters have been implicated in information gathering, each contributing through distinct mechanisms. Norepinephrine appears to facilitate information gathering by inducing hyper-reactivity to uncertainty. Dopamine seems to be particularly involved when information carries hedonic value, likely due to its role in reward processing. Finally, serotonin appears to constrain extended searches by amplifying the perceived cost of sampling.

## 5 Brain regions involved information gathering

Several brain regions have been implicated in processes that relate either directly or indirectly to active information gathering. These include:

1. Limbic areas such as amygdala and hippocampus, which are thought to play a role in statistical learning and uncertainty processing;
2. Insula, which is implicated in uncertainty representation and anticipation;
3. Fronto-striatal regions implicated in cost-benefit evaluation;
4. Parietal cortex, implicated in the representation of the value of information gain.

In this section, we will discuss how these regions can support information gathering based on their functional attributes described in the literature.

### 5.1 Limbic system: hippocampus and amygdala

In addition to its well-established role in memory and spatial cognition (Bird and Burgess, 2008; Burgess et al., 2002; Eichenbaum et al., 1999; T. Hartley et al., 2014), the hippocampus has increasingly been implicated in decision-making and uncertainty processing (Attaallah et al., 2024; Barron et al., 2013; Biderman et al., 2020; Enkavi et al., 2017; Palombo et al., 2015a, 2015b; Wimmer and Shohamy, 2012). These functions likely contribute to active information gathering.

Findings from animal studies link the hippocampus to *vicarious trial and error* (VTE) behaviour (Redish, 2016). Rats navigating maze tasks pause at decision points, scanning their surroundings before proceeding (Tolman, 1939; Tolman, 1948). This behaviour is thought to reflect deliberative processing of uncertainty, where animals search and evaluate potential trajectories before committing to an action (Gilbert and Wilson, 2007; Keller et al., 2020; Payne et al., 1993; Rangel et al., 2008). This process shares key mechanistic components with active information gathering (Keller et al., 2020; Redish, 2016), as both require the evaluation of possible future states to guide decision-making. The hippocampus is crucial in this context due to its established role in mental time travel–reconstructing past experiences (memory retrieval) and simulating future events (prospection and imagination) (Buckner and Carroll, 2007; Gilbert and Wilson, 2007; Schacter et al., 2007; Schacter et al., 2012; Schacter et al., 2017). Through this mechanism, animals are thought to mentally construct and evaluate potential routes before making a choice (Redish, 2016).

Hippocampal place cells in rodents have been shown to encode future trajectories of planned movements toward goals and rewards (Freyja et al., 2015; Pfeiffer and Foster, 2013).

Moreover, rats with hippocampal lesions display deficits in detecting and evaluating changes in reward contingencies within their environment (Bett et al., 2015), a finding recently extended to humans with hippocampal damage (Attaallah et al., 2024). This goal-directed decision-making process is thought to depend on interactions between the hippocampus and reward-processing regions, including the striatum and prefrontal cortex (PFC), in both animals and humans (Hassabis et al., 2007; Lebreton et al., 2013; Peters and Büchel, 2010; Schacter et al., 2012; Spiers and Gilbert, 2015; Wang et al., 2015).

In humans, numerous studies have highlighted the hippocampus’s role in uncertainty processing and valuation. For instance, hippocampal activation has been shown to correlate with the degree of sensory entropy (uncertainty) in presented stimuli when making decisions based on these cues (Harrison et al., 2006; Rigoli et al., 2019; Strange et al., 2005; Tobia et al., 2012). Other investigations have demonstrated the hippocampus’s contribution to uncertainty resolution and valuation, particularly in inferring and constructing the value of novel stimuli based on prior experiences that link to them (Barron et al., 2013; Wimmer and Buchel, 2016; Wimmer and Shohamy, 2012). This hippocampus-dependent valuation is also evident in reward-based decisions that require future episodic thinking and deliberation. Recent findings indicate that BOLD hippocampal signals correlate with deliberation time before making decisions (Bakkour et al., 2019) and that hippocampal dysfunction disrupts reward processing under uncertainty (Attaallah et al., 2024). These findings align with studies implicating the hippocampus in delay discounting, where the perceived value of future rewards increases with the availability of episodic details (Kwan et al., 2012; Peters and Büchel, 2010).

This utilisation of mental time travel not only aids inferential value processing but may also contribute to stabilising preferences and orienting individuals within value space. For example, one study found that patients with hippocampal damage exhibited deficits in transitive inference, leading to volatile and inconsistent preferences compared to matched controls (Enkavi et al., 2017). Similarly, hippocampal patients performing the Iowa Gambling Task struggled to develop preferences for advantageous card decks (Gupta et al., 2009; Gutbrod et al., 2006). In visual search tasks, these patients also displayed less consistent patterns of information gathering, suggesting increased stochasticity and an impaired ability to develop optimal search trajectories (Lucas et al., 2019). Moreover, hippocampal patients were found to be less efficient and prone to over-sampling compared to matched controls (Attaallah et al., 2024). Conversely, hyperactivation of hippocampal networks may heighten sensitivity to uncertainty, driving excessive information sampling without necessarily impairing sampling efficiency (Attaallah et al., 2022). Crucially, these deficits were independent of memory impairment or general reasoning abilities, indicating a distinct mechanism by which the hippocampus contributes to information seeking and uncertainty resolution.

A detailed mechanistic account of the hippocampus’s contribution to active sampling remains to be established. The hippocampus may be involved in multiple aspects of this process, including economic functions such as valuing samples and outcomes that agents seek when gathering information, as well as inferential and statistical roles in assessing uncertainty and generating and evaluating potential sampling trajectories to achieve a goal.

The amygdala’s role in information gathering is likely more straightforward, stemming from its contribution to uncertainty representation and modulation of affective and behavioural responses (Morriss et al., 2019; Tanovic et al., 2018). Along with the insula, the amygdala is consistently implicated in tasks and decisions requiring uncertainty processing. The uncertainty and anticipation model of anxiety (Grupe and Nitschke, 2013) proposes that the amygdala is a key region involved in modulating anticipatory responses to uncertainty. Through interactions with other brain regions involved in cost and probability estimation of uncertainty, such as the dorsomedial prefrontal cortex (dMFPC), orbitofrontal cortex (OFC), and insular cortex (IC), the amygdala is thought to increase vigilance and behavioural reactivity in the face of uncertainty (Grupe and Nitschke, 2013; Kim et al., 2011; Sarinopoulos et al., 2010; FeldmanHall et al., 2019). Mechanistically, the amygdala’s role is linked to its contribution to associative learning and conditioning, especially for negative and aversive cues (Amorapanth et al., 2000; Holland and Gallagher, 1999; Li et al., 2011; Moscarello and LeDoux, 2013). Previous studies have shown that amygdala activation correlates with expected loss when making decisions (De Martino, et al., 2010; Yacubian et al., 2006). Additionally, amygdala BOLD signals correlate with subjective reports of intolerance to uncertainty in both healthy controls and individuals with anxiety disorders (Morriss et al., 2015; Schienle et al., 2010; Somerville et al., 2013; Tanovic et al., 2018). These findings suggest that increased sampling behaviour in anxiety and related disorders (Attaallah et al., 2024; Sternheim et al., 2011) may be attributed to amygdalar dysfunction, promoting hypersensitivity to uncertainty and its prospective costs. This relationship between the amygdala and uncertainty processing provides insight into the neural mechanisms underlying information-gathering behaviours and their potential dysregulation in anxiety disorders.

### 5.2 The insula and other cortical regions

The insular cortex (IC), particularly its anterior portion, is perhaps the most consistently implicated region in decision-making under uncertainty and information gathering (Morriss et al., 2019). Numerous fMRI studies have reported insular activation in response to uncertainty across various task conditions. For instance, research has demonstrated that anticipation of unpredictable negative cues is associated with bilateral insular activity, an effect linked to higher anxiety scores and perceived uncontrollability (Alvarez et al., 2015). These findings align with the anticipatory model of anxiety, which posits that the anterior insula plays a crucial role in anticipating and estimating uncertainty and its associated costs (Grupe and Nitschke, 2013; Tanovic et al., 2018).

More direct investigations of information gathering, utilising the beads task, have shown that insular BOLD signals are modulated by both expected reward gain (Furl and Averbeck, 2011) and the degree of uncertainty (Krug et al., 2014). A recent study revealed that enhanced insular-hippocampal connectivity in subjective cognitive impairment (SCI) correlated with more urgent sampling behaviour to resolve uncertainty before decision-making (Attaallah et al., 2022). Functionally, the insula integrates affective and interoceptive signals with cognitive computations, supporting the assignment of salience and the anticipation of cost. These functions are shaped by dense serotonergic and noradrenergic innervation, which modulate aversive prediction and arousal-related responses to uncertainty (Benarroch, 2019; Uddin et al., 2017).

These functions of the insula may be attributed to its rich structural and functional connections with other cortical regions involved in probability and value computation, such as the parietal and prefrontal cortices (Craig, 2009; Namkung et al., 2017; Uddin et al., 2017). Most studies investigating neural correlates of information gathering report co-activation of these regions along with the IC (Banca et al., 2016; Chang et al., 2013; Furl and Averbeck, 2011; Paulus and Stein, 2006; Pavuluri and May, 2015; Shadlen and Newsome, 2001). For example, one study demonstrated that parietal BOLD signals were greater in participants who collected more evidence before making decisions (Furl and Averbeck, 2011). Additionally, the number of beads drawn in another study correlated with the volumes of the inferior parietal region and dorsolateral prefrontal cortex (dlPFC) (Banca et al., 2016).

The parietal cortex, particularly the intraparietal sulcus and inferior parietal lobule, plays a central role in representing accumulated evidence and computing the instrumental value of additional information. Early neurophysiological studies in non-human primates demonstrated that neurons in these regions integrate sensory evidence during dynamic decision-making tasks, independent of immediate reward signals (Shadlen and Newsome, 2001). More recent human work has confirmed that the parietal cortex tracks the evolving precision of beliefs and encodes information gain related to uncertainty reduction (Horan et al., 2019).

The dorsolateral prefrontal cortex (dlPFC) is another key contributor to information gathering, implicated in the executive control of deliberation, planning, and response inhibition (Badre and Nee, 2018; Friedman and Robbins, 2021; Mushtaq et al., 2011). It is thought to support the maintenance of task goals and the suppression of premature responses, particularly under conditions of high uncertainty or distraction (Miller and Cohen, 2001; Sakai et al., 2002). A key subcortical connection of the dlPFC includes the subthalamic nucleus (STN), stimulation of which has been shown to induce decisional impulsivity and reduced evidence accumulation on the beads task (Voon et al., 2017). This finding underscores the importance of the dlPFC-STN network in regulating cognitive control mechanisms that delay premature commitments in favour of more deliberate sampling. Notably, while STN stimulation altered decision thresholds, it did not significantly affect confidence ratings, suggesting a dissociation between decision formation and metacognitive monitoring. The latter appears to be more closely associated with medial prefrontal structures, particularly the dorsal medial prefrontal cortex (dmPFC), which may help integrate accumulated evidence with subjective appraisal of uncertainty.

The dorsal medial prefrontal cortex (dmPFC) plays a distinct yet complementary role in active information gathering, primarily through its involvement in metacognitive control, cognitive conflict monitoring, and the regulation of urgency signals. This region is typically engaged when individuals face high-conflict decisions, where multiple competing alternatives require the resolution of internal uncertainty and adjustment of decision policies (Botvinick et al., 2004; Ridderinkhof et al., 2004; Shenhav et al., 2016). The dmPFC may thus encode a form of urgency signal that increases over time, dynamically modulating the trade-off between information accumulation and commitment to action (Pisauro et al., 2017; Thura et al., 2012). This mechanism may serve to prevent excessive deliberation, particularly in contexts where continued sampling incurs escalating opportunity costs. Additionally, the dmPFC is increasingly recognised as a critical hub for metacognitive control, coordinating behavioural adjustments based on internally generated evaluations of confidence and task performance (Botvinick et al., 2004; Shenhav et al., 2016; Su et al., 2022; Yuki et al., 2024). While ventro-medial prefrontal cortex (vmPFC) activity has been linked to meta-cognitive monitoring and the representation of subjective value and affect (De Martino, et al., 2013; Lebreton et al., 2015), the dmPFC appears to translate these self-evaluative signals into executive modulation of sampling strategies and response thresholds. This function likely interfaces with its broader involvement in cost-benefit integration (Bartra et al., 2013; Ciaramelli et al., 2021; Hogan et al., 2018; Levy and Glimcher, 2012), a topic further elaborated in the following section.

Collectively, these findings suggest that information gathering is subserved by a distributed fronto-parietal-insular network, wherein interoceptive salience (insula), evidence accumulation (parietal cortex), strategic control (dlPFC), and metacognitive arbitration (dmPFC) are dynamically coordinated to guide adaptive sampling decisions under uncertainty.

### 5.3 Valuation system brain regions

A substantial body of literature has highlighted the crucial role of reward-related regions in various aspects of value-based decision making. These regions are thought to function in coordination, forming what has been referred to as the ‘valuation/reward system’ (Bartra et al., 2013; Haber, 2017; Haber and Knutson, 2009). This network includes cortical and subcortical regions such as the orbitofrontal cortex (OFC), vmPFC, and ventral striatum (VS), which share strong connections with brain regions implicated in uncertainty processing, such as the amygdala and insula (Grupe and Nitschke, 2013; Haber, 2017; Haber and Knutson, 2009; Kim et al., 2011; Morriss et al., 2015; Morriss et al., 2019; Uddin et al., 2017). Most of these reward-related brain structures are also implicated in cost representation, as suggested by neurophysiological and neuroimaging evidence in animals and humans (Chen, 2021).

Gathering information is often a costly process that might involve several forms of cost such as time, effort, money, and cognitive energy (Hauser et al., 2017; Hauser et al., 2018; Juni et al., 2016). These different forms of costs might be preferentially encoded by different regions. For example, the OFC has been linked to temporal cost encoding, with animals and humans with OFC lesions demonstrating stronger temporal discounting and a preference for immediate rewards (Bechara et al., 2000; Berlin et al., 2004; Mobini et al., 2002; Rudebeck et al., 2006). The anterior cingulate cortex, on the other hand, has been linked to effort costs in goal-directed decision making (Croxson et al., 2009; Pessiglione et al., 2018). However, other studies suggest a degree of overlap in the representations of cost and value, promoting a view that some of these regions are globally involved in value and cost representation regardless of the identity of the stimuli (Carlezon and Thomas, 2009; Gläscher et al., 2009; Monosov, 2017; Ruff and Fehr, 2014; Vassena et al., 2014).

Regardless of the exact neural mechanism, it is likely that the expected cost (and hedonic value) of a sample during information gathering is represented in a similar fashion in these brain regions.

Crucially, the function of the valuation network is not limited to these representations. Through their extensive connections with other brain networks, including limbic, para-limbic, and cortical systems, regions in the network such as the prefrontal cortex might integrate and compare value utilising information supplied from these sources (Chen, 2021; Rangel et al., 2008). This process is essential for cost-benefit evaluation, resulting in a subjective value assignment that ultimately drives decision making. One study has indeed shown that differences between information value and cost correlate with activity in the prefrontal cortex (Basten et al., 2010). Intriguingly, this signal, as it accumulates, was found to correlate with parietal cortex activity, which, as discussed previously, might serve to track accumulation of information utility (Horan et al., 2019).

Thus, regions in the valuation system such as the OFC and ACC might serve to support cost-benefit valuation of the sampling process by contributing to cost processing and subjective value computation.

**Fig. 3:**
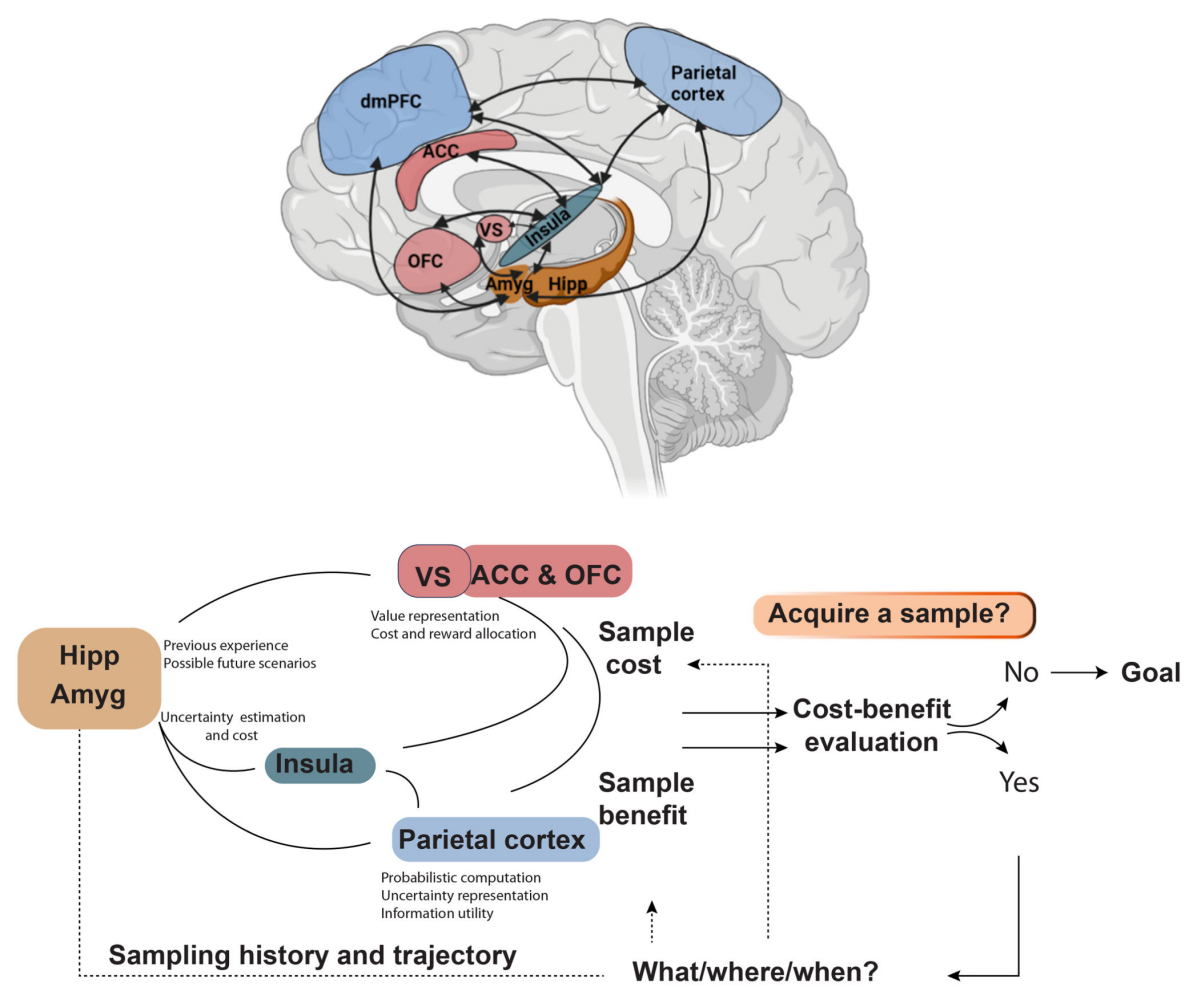
Neuroanatomical correlates of active information gathering. Active information gathering engages a distributed network of brain regions involved in valuation, decision-making, learning, and uncertainty monitoring. The process requires neuroeconomic computations that weigh the potential benefits of acquiring additional information against the costs of continued sampling, in order to determine an optimal stopping point and maximise expected value (see Fig. 2). Estimating the benefit of further sampling entails evaluating environmental uncertainty and simulating possible information trajectories. These computations have been linked to limbic regions such as the amygdala and hippocampus, and to the insula, which is thought to encode interoceptive signals and uncertainty-related salience. These areas interact with the parietal cortex, implicated in tracking the instrumental value of information and guiding attention toward informative cues. The dorsomedial prefrontal cortex (dmPFC) plays a central integrative role, supporting prospective inference and cognitive control over exploratory decisions. It is implicated in estimating control demand, predicting future decision difficulty, and balancing competing goals under uncertainty. Reward-related regions,including the ventral striatum (VS), orbitofrontal cortex (OFC), and anterior cingulate cortex (ACC), contribute to estimating sampling costs (e.g., effort, time, opportunity cost) and integrating these with expected informational value to guide action selection. Together, these interconnected regions form a circuit that supports adaptive information sampling under uncertainty. Abbreviations: Hipp, Hippocampus; Amyg, Amygdala; VS, Ventral Striatum; ACC, Anterior Cingulate Cortex; OFC, Orbitofrontal Cortex; dmPFC, Dorsomedial Prefrontal Cortex.

Overall, as shown in Fig. 3, information sampling to resolve uncertainty is a complex behavioural and cognitive operation that recruits several cortical and subcortical regions at different stages to perform cost-benefit optimisation underlying sampling decisions.

## 6 Conclusions and future directions

Active information gathering is a fundamental cognitive process that enables organisms to navigate uncertainty and make adaptive decisions. This review has synthesised current knowledge on the behavioural, neural, and computational mechanisms underlying information sampling across health and disease. Several key themes have emerged from this analysis. Firstly, information gathering behaviour varies across clinical conditions, with some disorders associated with under-sampling (e.g. schizophrenia, addiction) and others with over-sampling (e.g. obsessive-compulsive disorder) (Table 1). This highlights the potential of information sampling paradigms as transdiagnostic markers of psychopathology. Secondly, multiple neurotransmitter systems modulate information gathering, including norepinephrine, dopamine, and serotonin. These systems appear to influence different aspects of the sampling process, such as urgency, reward valuation, and cost perception. A distributed network of brain regions supports information gathering, including limbic areas (hippocampus, amygdala), insula, fronto-striatal circuits, and parietal cortex. These regions contribute to uncertainty estimation, value computation, and decision-making under uncertainty (Fig. 3).

Computational approaches have provided valuable insights into the cognitive processes underlying information gathering, framing it as a cost-benefit optimisation problem (Fig. 2). This framework allows for more precise quantification of the mechanisms that may be disrupted in clinical populations. The hippocampus has emerged as a key region of interest, with recent evidence suggesting it plays a crucial role in decision-making under uncertainty and information sampling behaviour. This expands our understanding of hippocampal function beyond its traditional roles in memory and spatial cognition.

Building on the findings synthesised in this review, several promising avenues for future research emerge. Longitudinal studies are needed to determine whether aberrant information gathering behaviours precede the onset of clinical symptoms, potentially serving as early markers of vulnerability to psychiatric disorders. Such studies could help elucidate the causal relationships between information sampling deficits and the development of various psychopathologies. Further development and application of computational models to clinical populations may help elucidate the specific cognitive processes disrupted in different disorders, potentially informing more targeted interventions. These models could be refined to incorporate additional factors such as metacognitive processes and the influence of prior beliefs, providing a more nuanced understanding of information gathering deficits across various conditions.

A comprehensive examination of how information gathering behaviour evolves across the lifespan, from early childhood to older adulthood, could provide valuable insights into both typical and atypical cognitive development. This developmental perspective may shed light on critical periods for the emergence of adaptive information sampling strategies and potential windows for intervention in clinical populations. Future studies should aim to combine behavioural, computational, neuroimaging, and pharmacological approaches to develop a more comprehensive understanding of the mechanisms underlying information gathering. This multi-modal approach could help clarify the specific contributions of different neurotransmitter systems and brain regions to various aspects of the sampling process, such as uncertainty estimation, cost-benefit evaluation, and decision threshold setting. There is potential to develop novel cognitive training paradigms or pharmacological interventions targeting specific aspects of information gathering behaviour, based on the insights gained from basic science research. For example, interventions aimed at modulating noradrenergic or serotonergic function could be explored as potential treatments for disorders characterised by aberrant sampling behaviour.

Development of more naturalistic information sampling paradigms that better reflect real-world decision-making scenarios could enhance the clinical relevance and applicability of findings. This could involve the use of virtual reality environments or ecological momentary assessment techniques to capture information gathering processes in daily life. Finally, further exploration of how personality traits, cognitive abilities, and environmental factors influence information gathering strategies could provide insights into why some individuals are more vulnerable to maladaptive decision-making patterns. This line of research could inform personalised approaches to both assessment and intervention in clinical populations.

By pursuing these research directions, we can deepen our understanding of how humans and other animals navigate uncertainty through active sampling of their environment. This knowledge has broad implications for cognitive science, neuroscience, and clinical practice, potentially informing the development of novel diagnostic tools and therapeutic approaches for a range of psychiatric and neurological conditions.

## Figures and Tables

**Table 1:** Information gathering in different conditions.

Condition	Sampling behaviour (vs controls)	Proposed Mechanism	Examples of relevant studies
Delusional disorders (e.g., psychosis and schizophrenia)	Under-sampling	Cognitive rigidity in belief formation Lower decision thresholds Sampling cost over-estimation	Ermakova et al., 2019; Garety et al., (2005); Huq et al., (1988); McLean et al., (2016)
Obsessive compulsive disorder (OCD)	Over-sampling	Deficit in subjective uncertainty estimation Dissociation between confidence and action Delayed emergence of sampling cost signals and urgency	Hauser et al.,(2017); Hauser et al., (2018)
Addiction (alcohol, cocaine, pathological gaming)	Under-sampling	Hypersensitivity to reward Enhanced valuation of uncertain options with higher rewards	Banca et al., (2016); Clark et al., (2006) Irvine et al., (2013); Joos et al., (2013)
Impulsivity (mainly PD patients)	Under-sampling	Reduced sensitivity to uncertainty Deficits in probabilistic reward mapping	Averbeck et al., (2013); de Rezende Costa et al., 2016 Djamshidian et al., (2012); Kehagia et al., (2014)
Anxiety and depression	Over-sampling	Exaggerated estimation of uncertainty Intolerance of uncertainty Reduced reward valuation	Attaallah et al., (2022); Crockett et al., (2012); Hildebrand-Saints and Weary, (1989)
Alzheimer’s disease (AD)	Under-sampling	Impaired uncertainty processing and estimation Less aversion to uncertainty and ambiguity	Sinz et al., (2008); Zamarian et al., (2015)
